# miRNA Signatures in Sera of Patients with Active Pulmonary Tuberculosis

**DOI:** 10.1371/journal.pone.0080149

**Published:** 2013-11-21

**Authors:** Paolo Miotto, Grace Mwangoka, Ilaria C. Valente, Luca Norbis, Giovanni Sotgiu, Roberta Bosu, Alessandro Ambrosi, Luigi R. Codecasa, Delia Goletti, Alberto Matteelli, Elias N. Ntinginya, Francesco Aloi, Norbert Heinrich, Klaus Reither, Daniela M. Cirillo

**Affiliations:** 1 Emerging Bacterial Pathogens Unit, Division of Immunology, Transplantation and Infectious Diseases, San Raffaele Scientific Institute, Milan, Italy; 2 Ifakara Health Institute-Bagamoyo Research and Training Centre, Bagamoyo, United Republic of Tanzania; 3 Clinical Epidemiology and Medical Statistics Unit, Department of Biomedical Sciences, University of Sassari; Research, Medical Education and Professional Development Unit, AOU Sassari, Sassari, Italy; 4 Vita-Salute San Raffaele University, Milan, Italy; 5 Regional Reference Center for TB “Villa Marelli”, Niguarda Ca’ Granda Hospital, Milan, Italy; 6 Translational research Unit, National Institute for Infectious Diseases “L. Spallanzani”, Rome, Italy; 7 Institute of Infectious and Tropical Diseases, World Health Organization Collaborating Centre for TB/HIV co-infection, Brescia University, Brescia, Italy; 8 National Institute of Medical Research-Mbeya Medical Research Centre, Mbeya, United Republic of Tanzania; 9 St. Francis Nsambya Hospital/AISPO, Kampala, Uganda; 10 Italian Association for Solidarity Among People (AISPO NGO), Kampala, Uganda; 11 Division for Infectious Diseases and Tropical Medicine, Ludwig Maxmillian University, Munich, Germany; 12 Swiss Tropical and Public Health Institute and University of Basel, Basel, Switzerland; South Texas Veterans Health Care System and University Health Science Center San Antonio, United States of America

## Abstract

Several studies showed that assessing levels of specific circulating microRNAs (miRNAs) is a non-invasive, rapid, and accurate method for diagnosing diseases or detecting alterations in physiological conditions. We aimed to identify a serum miRNA signature to be used for the diagnosis of tuberculosis (TB). To account for variations due to the genetic makeup, we enrolled adults from two study settings in Europe and Africa. The following categories of subjects were considered: healthy (H), active pulmonary TB (PTB), active pulmonary TB, HIV co-infected (PTB/HIV), latent TB infection (LTBI), other pulmonary infections (OPI), and active extra-pulmonary TB (EPTB). Sera from 10 subjects of the same category were pooled and, after total RNA extraction, screened for miRNA levels by TaqMan low-density arrays. After identification of “relevant miRNAs”, we refined the serum miRNA signature discriminating between H and PTB on individual subjects. Signatures were analyzed for their diagnostic performances using a multivariate logistic model and a Relevance Vector Machine (RVM) model. A leave-one-out-cross-validation (LOOCV) approach was adopted for assessing how both models could perform in practice. The analysis on pooled specimens identified selected miRNAs as discriminatory for the categories analyzed. On individual serum samples, we showed that 15 miRNAs serve as signature for H and PTB categories with a diagnostic accuracy of 82% (CI 70.2–90.0), and 77% (CI 64.2–85.9) in a RVM and a logistic classification model, respectively. Considering the different ethnicity, by selecting the specific signature for the European group (10 miRNAs) the diagnostic accuracy increased up to 83% (CI 68.1–92.1), and 81% (65.0–90.3), respectively. The African-specific signature (12 miRNAs) increased the diagnostic accuracy up to 95% (CI 76.4–99.1), and 100% (83.9–100.0), respectively. Serum miRNA signatures represent an interesting source of biomarkers for TB disease with the potential to discriminate between PTB and LTBI, but also among the other categories.

## Introduction

Tuberculosis (TB) remains one of the most relevant infectious diseases with nearly 9 million cases and 1.4 million deaths per year worldwide [1]. Due to the complexity of the clinical presentations of the infection caused by members of the *Mycobacterium tuberculosis* complex (latent asymptomatic infection, active pulmonary and/or extra-pulmonary disease), accurate classification of cases is essential to address the most appropriate clinical management [2]–[4].

Current standards for TB diagnosis, including the most sensitive molecular tests, rely on the detection of the pathogen, thus being dependent on the bacterial load in the specimen analyzed; indeed, diagnosing TB in children is a difficult task because the mycobacterial load is often low [5]–[7]. Similarly, extra-pulmonary TB (EPTB) cases are often challenging to diagnose due to the difficulties in obtaining samples for microbiological investigations, and are affected by unpredictable distribution of bacteria in tissues. Therefore, EPTB and smear-negative pulmonary TB (PTB) are usually diagnosed *ex juvantibus*. Finally, despite the importance of utterly discriminating between latent TB infection (LTBI) and active TB, clear-cut biological markers separating the two conditions are not yet available [8]–[10]. Biomarkers and surrogate endpoints are therefore crucial tools for the development of innovative strategies for TB management [11].

The development of diagnostic tests based on host biomarkers is advocated to clinically categorize paucibacillary or not microbiologically-confirmed TB cases, and for proper identification of LTBI cases [11], [12]. The need for biomarkers extends beyond the urgency for improved diagnostic tools: the assessment of the disease status and of the risk of progression to active disease or the assessment of treatment success early during therapy are critical bottlenecks in the development of new vaccines and drugs, and could allow categorization of therapy based on individual risk of unfavorable outcome.

The ideal biomarker should be a stable molecule, and should be in sufficient amounts for easy detection in accessible body fluids [9], [13]. The discovery that human microRNAs (miRNAs) expression is frequently altered in various diseases has uncovered a new repertoire of molecular factors, which warrants investigation to further elucidate their role in physiology and disease [14]. In the cell, miRNAs post-transcriptionally regulate the expression level of target genes [15]. The miRNA highly-conserved regulatory system does not remain confined to the intracellular compartment: they can be transferred via body fluids (including plasma, serum, urine, and saliva), thus modulating translational responses by intercellular communication [16]–[19].

miRNAs could represent an ideal biomarker, owing to the sampling easiness, the inherent stability and the resilience [19], [20]. However, their characterization during viral or bacterial infection has raised interest only recently, and data on TB remain limited to few studies [21]–[23].

This research aimed at identifying miRNA profiles associated with the different phases of *M. tuberculosis* infection (PTB, EPTB, LTBI), and non-tubercular lung infections as well as in healthy condition and showing their use as specific signatures for PTB diagnosis.

## Materials and Methods

### Ethical Statements

The protocol of the study was approved by the Ethical Committee of the San Raffaele Scientific Institute, Milano, Italy (GO/URC/ER/mm prot. N. 82/DG) and of the participating institutions in Uganda and Tanzania. The study was conducted in full compliance with the principles of the Declaration of Helsinki. All samples were collected from individuals who had signed an informed consent form for the purpose of the study and for cryopreservation of their biological samples.

### Study Population

The following case definitions were adopted to categorize individuals enrolled in the study:

- Patients with active pulmonary TB (PTB): sputum smear microscopy positive for acid-fast bacilli, *M. tuberculosis* complex culture and/or Xpert MTB/RIF (Cepheid, Sunnyvale, CA) positive patients with pulmonary disease, HIV negative individuals;
- Patients with active pulmonary TB and HIV co-infection (PTB/HIV): sputum smear microscopy positive for acid-fast bacilli, *M. tuberculosis complex* culture and/or Xpert MTB/RIF positive patients with pulmonary disease, HIV positive confirmed cases;
- Patients with active extra-pulmonary TB (EPTB): culture positive TB cases with any extra-pulmonary disease localization, HIV negative individuals;
- Latent TB infection cases (LTBI): subjects who resulted interferon-γ release assay (IGRA) or tuberculin skin test (TST) positive with no signs/symptoms of active disease;
- Subjects affected by pulmonary infectious diseases other than TB (OPI): clinical diagnosis (clinical exam plus imaging), with or without microbiological confirmation, IGRA and/or TST negative;
- Healthy subjects (H): IGRA and/or TST negative, without any known risk factors for LTBI, without any clinically relevant conditions.

The study populations of adult subjects were enrolled between September 2009 and December 2012 from two studies, namely *TBnew* and *TB CHILD*.

#### TBnew

Subjects enrolled at the San Raffaele Hospital (Milano, Italy), Spedali Civili of Brescia (Brescia, Italy), Regional Center for TB “Villa Marelli”, Niguarda Hospital (Milano, Italy), and at the National Institute for Infectious Diseases “L. Spallanzani” (Roma, Italy) belonged to the following categories: PTB, EPTB, LTBI, H, OPI.

#### TB CHILD

Subjects enrolled at Ifakara Health Institute - Bagamoyo Research and Training Centre (BRTC) (Pwani, Tanzania), NIMR-Mbeya Medical Research Programme (MMRC) (Mbeya, Tanzania), and at the St. Francis Nsambya Hospital (Kampala, Uganda) belonged to the following categories: PTB, PTB/HIV, H.

All subjects included in the study underwent the following procedures:

- phlebotomy through a 21G butterfly device to minimize hemolysis during specimen collection. Additive-free blood collection tubes were chosen to minimize unwanted modification of miRNA content in the serum;
- collection of clinically relevant data to determine TB status (with relevant confirmatory exams), and HIV status. A questionnaire to capture any additional information such as: pregnancy, smoking, current medical problems (diabetes, transplant, silicosis, sarcoidosis, cancer), current therapies with particular focus on immunosuppressive, antiretroviral and anti-TB ones was compiled for all patients during enrolment.

All information was stored in an electronic data-protection system.

**Figure 1:**
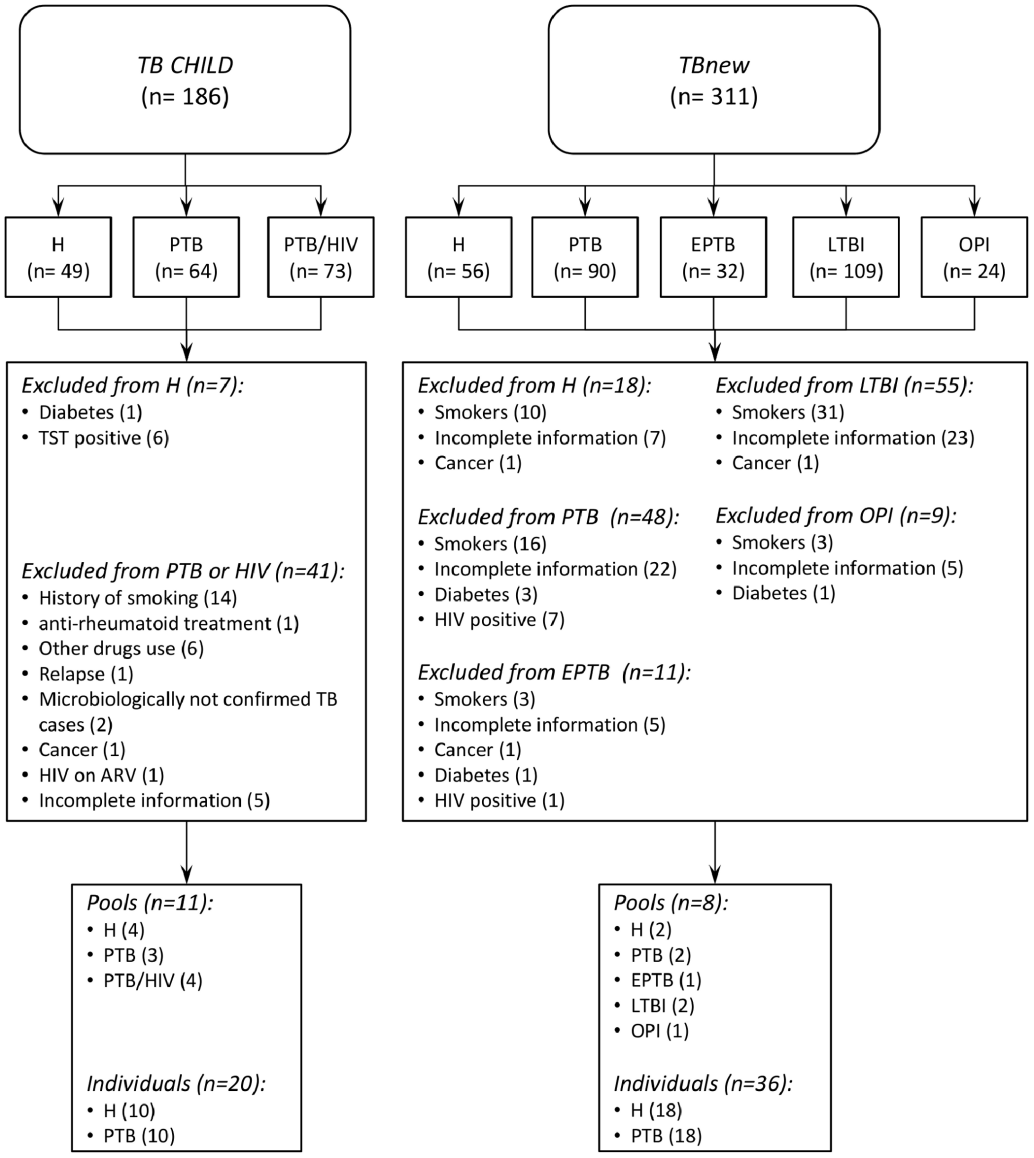
Criteria for the inclusion in the study population.

Enrolment and exclusion criteria of the study population are summarized in Figure 1.

### Serum Preparation

Within 4 hours from time of phlebotomy, after coagulation, tubes were centrifuged at 2500 rpm for 10 minutes, with separation of serum from corpuscular fractions. Subsequently, serum fraction was transferred – in sterile environment – into 15 mL tubes and underwent a second centrifugation at 2800 rpm for 10 minutes, in order to achieve maximum removal of cellular component. Serum was hence transferred in aliquot of 1 mL into cryogenic vials and stored at −80°C. For RNA extraction, sera were thawed on ice and the degree of hemolysis was determined through spectrometry analysis of free hemoglobin as previously described [24]. A cut-off value of hemoglobin concentration of >10 mg/dL was considered for hemolyzed samples.

### Serum Pools

Each pool for qRT-PCR analysis was composed of 10 non-smoker subjects from the same category, equally distributed by gender. Selected subjects were free of co-morbidities (as for the pathologies investigated during the enrolment interview) to avoid confounding effects on miRNA profiles. A serum aliquot from each subject was thawed on ice, and 500 µL of serum from each sample were mixed together in order to obtain a homogeneous pooled serum sample. One mL of pooled serum was used for RNA extraction and subsequent miRNA analysis in duplicate.

### Serum from Single Individuals

To refine the results obtained from pooled sera, we performed individual serum analysis on 18 subjects belonging to the PTB and H categories from the *TBnew* group, and on 10 subjects from the *TB CHILD* group. As for pooled sera, individuals were selected free of co-morbidities among non smokers. A serum aliquot from each subject was thawed on ice, and 1 mL of serum from each individual was used for RNA extraction and subsequent miRNA analysis.

### RNA Extraction

RNA extraction was performed using the mirVana miRNA isolation kit (Life Technologies) according to the manufacturer’s instructions for isolating total RNA. RNA samples were stored at −80°C until use.

### Serum miRNA Profiling

Serum miRNAs analysis was performed with TaqMan® Low Density Array (TLDA) Human MicroRNA Panels A and B, investigating an overall of 671 different miRNAs (Life Technologies). Retro-transcription was performed with TaqMan® microRNA reverse transcription kit and MegaPlex RT Primers Human Pool A v2.1 and B v2.0 components (Life Technologies), according to the manufacturer’s instructions. For each RT reaction 15 ng of total RNA were used. After RT step, samples were pre-amplified using the MegaPlex PreAmp Primers A and B and the TaqMan® PreAmp Mater Mix according to the manufacturer’s instructions (Life Technologies). TLDAs were performed using a 7900HT Fast Real-Time PCR System or a ViiA™ 7 Real-Time PCR System according to manufacturer’s instructions (Life Technologies). On pooled sera, we performed analyses in biological and technical replicate for each array panel (A and B), whereas for each individual sera we performed single arrays (panels A+B).

### Data Analysis and Normalization

Data from TaqMan® were collected with SDS v2.4.1, ViiA™ 7 v1.1, and RQ Manager v1.2.2 software (Applied Biosystems), (baseline: automatic; threshold: 0.20; maximum allowable C_t_: 35.0). Data analysis was performed in R and Bioconductor environment [25]. We first preprocessed raw C_t_ values by means of quantile normalization, as described elsewhere [26]–[28]. This widely used approach is based on the assumption that only few miRNAs are differentially expressed. As a general result, this method provides homogenous data with the same distribution and the correlation coefficient between observations increases compared to raw data. Normalized data distribution was graphically inspected.

### Statistical Analysis

Results from pools and individuals were analyzed separately.

#### Pools

We performed one-to-one category comparisons between mean C_t_ values fitting a constrained regression model with MM robust estimators [29], [30]. These robust estimates have a high breakdown-point and are not affected by the presence of outliers or differently expressed miRNAs. We then computed the Empirical Distribution Function of residuals and filtered miRNAs associated with residuals outside the Inter Quartile range (*i.e.* outside the 1^st^ quartile –3^rd^ quartile interval) of the residuals distribution and we defined them as “interesting miRNAs” or “relevant miRNAs”. Circular visualization of data was made by Circos software [31].

#### Individuals

As our first step we filtered out miRNAs detected C_t_<35 in at least ≥80% of subjects of at least one of the categories considered (H in *TBnew*, PTB in *TBnew*, H in *TB CHILD*, and PTB in *TB CHILD*). For these filtered out miRNAs we performed a two ways ANOVA for health status and genetic makeup (defined as for country of birth). P-values were computed non-parametrically by means of permutations [32]. We checked for False Discovery Rate (FDR) with the method described by Benjamini and Yekutieli [33]. miRNAs showing both (i) an adjusted p-value (*p-adj*) <0.05 on individuals and (ii) relevant by pooled specimens analysis were considered for miRNA signature definition.

#### Performances of the signature

To assess the single miRNA performance in identifying health status a Receiver Operating Characteristic (ROC) curve based on kernel density distributions method fit as described in [34]. As overall measures of the performance in distinguish cases, the associated Area under the curve (AUC) was calculated and the p-values computed by means of permutations.

To assess and compare diagnostic performances of the miRNA signature identified, we fitted a multivariate logistic model selected by maximizing the Akaike Information Criteria (AIC) and a Relevance Vector Machine (RVM) model [35]–[37]. In contrast with Support Vector Machine, RVM follow a Bayesian approach giving *a posteriori* probability of the class. This makes the results from the two approaches more directly comparable. ROC curve and associated AUC were also computed for the logistic model.

A leave-one-out-cross-validation (LOOCV) approach was adopted for assessing how the results of both the RVM and AIC logistic regression predictive models would perform in practice. Performances were summarized in terms of sensitivity, specificity, positive predictive value (PPV), negative predictive value (NPV), and diagnostic accuracy.

## Results

### Study Population

**Figure 2:**
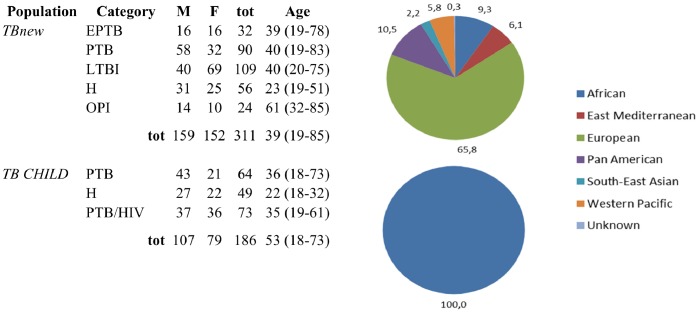
Demographic data of the *TBnew* and the *TB CHILD* populations. The Figure reports mean age for the categories of subjects enrolled and distribution of the country of birth in the different World Health Organization regions.

A total of 311 subjects (159 males, 51.2%) were enrolled within the *TBnew* group. As summarized in Figure 2 there were 56 healthy subjects, 109 individuals with LTBI (75 recent contacts of active PTB cases; 34 non-recent contacts), 24 subjects affected by other pulmonary infections (OPI), 32 EPTB and 90 PTB patients. Subjects were recruited in Italy but were born in countries located in the different World Health Organization (WHO) regions (Figure 2). The *TB CHILD* group includes only individuals from Tanzania and Uganda. A total of 186 subjects were enrolled (107 males, 57.5%): 49 healthy subjects, 64 PTB patients, and 73 patients with PTB and HIV co-infection. In the latter category the median CD4^+^ cell count was 198.4 cells/mL (interquantile range: 277.1) compared with 707.7 cells/mL (interquantile range: 813.3) of PTB subjects without HIV infection. H and PTB populations were statistically comparable for mean age values of enrolled subjects (p-values for H and PTB: 0.29, and 0.80, respectively). Eight pools from the *TBnew* group, and 11 pools from the *TB CHILD* group were considered for the analysis. We used a subset of individuals to refine the miRNA signatures identified on pooled specimens. Briefly, we performed individual serum analysis for 36 and 20 subjects for the *TBnew*, and the *TB CHILD* group, respectively (details are reported in Table S1).

### Normalization of qPCR Data

The TLDA is a 384-well microfluidic card containing dried TaqMan® primers and probes. Array A focuses on more highly characterized miRNAs while array B contains many of the more recently discovered miRNAs along with the miR* sequences. The use of two panels (array A and array B) enables quantitation of gene expression levels of up to 671 different miRNAs. This is accomplished by loading the cDNA product onto the array for PCR amplification and real-time analysis. MegaPlex Pools are designed to detect and quantitate up to 380 microRNAs (miRNAs) per pool in human species thanks to a set of stem-looped reverse transcription primers (MegaPlex RT Primers) that enable the simultaneous synthesis of cDNA and a set of miRNA-specific forward and reverse primers (MegaPlex PreAmp Primers) intended for use with very small quantities of starting material. The primers enable the unbiased preamplification of the miRNA cDNA target by PCR prior to loading the TaqMan® MicroRNA Array.

After the quantile normalization procedure, the C_t_ values of four miRNAs (ath-miR159a, MammU6, RNU44, and RNU48) detected by both array A and array B were compared. As showed in Table S2, C_t_ values were consistent between “pools – individuals”, “array A – array B”, and “*TBnew* – *TB CHILD*”. Normalized data from pooled and individual specimens are reported in Table S3.

### Analysis of Serum miRNA Profiles in Pooled Samples

Normalized qPCR data from pools showed 277 miRNAs undetectable in the categories (H, PTB, LTBI, OPI, EPTB, and PTB/HIV) from both groups (*TBnew*, and *TB CHILD*).

The mean C_t_ value for each miRNA was calculated and a one-to-one comparison between different categories was carried out. Residual values are available in Table S4.

**Figure 3:**
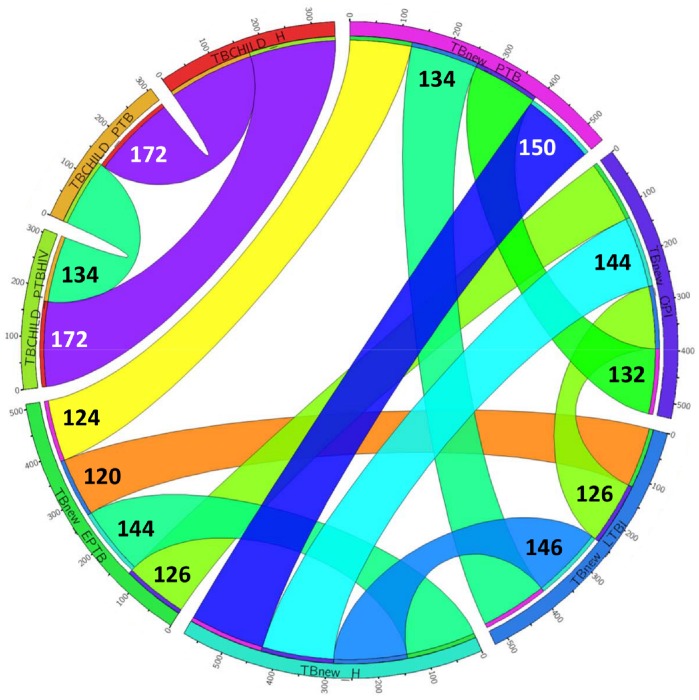
Summary of candidate serum miRNAs selected as relevant to discriminate among the categories according to the analysis of pooled specimens. Population and category of pooled specimens are reported on the circumference. The thickness of the ribbons connecting two categories is proportional to the number of miRNAs potentially interesting in the discrimination between the categories linked.

**Figure 4:**
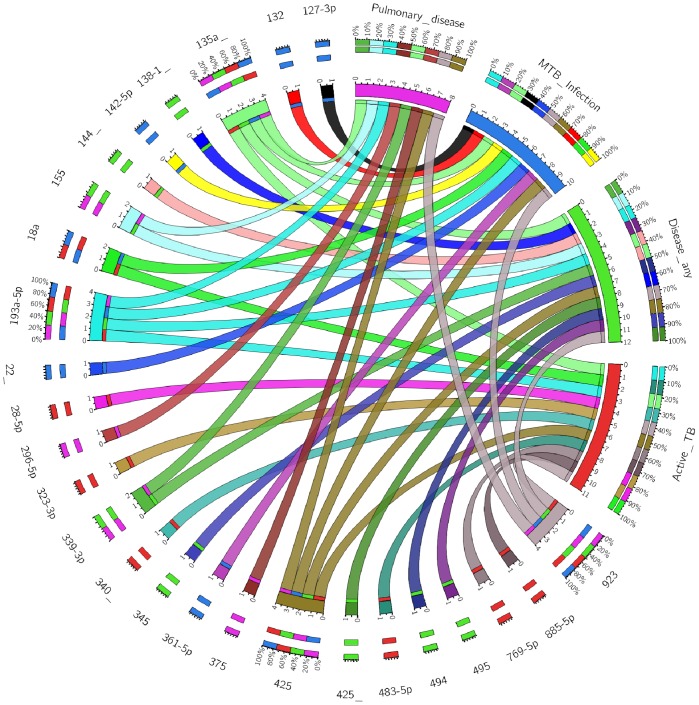
Summary of putative serum miRNA signatures discriminating among relevant clinical categories according to the analysis of pooled specimens. Clinical category and miRNAs are reported on the circumference. The ribbons represent the connection between miRNAs and clinical categories. The serum miRNAs have been defined by the following filters: Infection: H *vs* PTB, H *vs* LTBI, H *vs* EPTB, H *vs* PTB/HIV, OPI *vs* PTB, OPI *vs* LTBI, OPI *vs* EPTB; Active TB: H *vs* PTB, H *vs* EPTB, H *vs* PTB/HIV, OPI *vs* PTB, OPI *vs* EPTB, LTBI *vs* PTB, LTBI *vs* EPTB; Symptoms: H *vs* PTB, H *vs* OPI, H *vs* PTB/HIV, H *vs* EPTB, LTBI *vs* PTB, LTBI *vs* EPTB, LTBI *vs* OPI; Pulmonary disease (any): H vs PTB, H vs OPI, H vs PTB/HIV, LTBI vs PTB, LTBI vs OPI, EPTB vs PTB, EPTB vs OPI.

Figure 3 summarizes the number of miRNAs outside the 1^st^ and 3^rd^ quantile tails of the distribution of the residuals obtained by comparing two categories. The two percentiles considered, should most probably contain the miRNAs that are significantly different between the two compared categories. According to this qualitative analysis based on the distribution of the residuals, between 120 and 172 serum miRNAs could allow to discriminate among the categories considered in this study. For example, 134 miRNAs showed to be relevant in differentiating LTBI and PTB, whereas 132 miRNAs would allow discriminating between PTB and OPI. After filtering the pooled specimen results according to clinically relevant categories, we identified putative serum miRNA signatures defining MTB infection, active TB, pulmonary disease, or any of the disease statuses considered in this study (Figure 4).

To optimize our approach, for the PTB and H categories, we performed the analysis of individual sera.

### Analysis of Serum miRNA Profiles in Individuals

Serum miRNA profiles from 18 H, and 18 PTB from *TBnew* group as well as 10 H and 10 PTB from the *TB CHILD* group were analyzed.

Out of the 671 miRNAs tested, 126 targets were detected in 80% of the subjects in at least one of the analyzed categories. As reported in Table 1, 71 miRNAs showing a p-value <0.05 were identified: we could detect differences in serum miRNA profiles between the *TBnew* and *TB CHILD* individuals (35 miRNAs, 49.3%), between H and PTB (29 miRNAs, 40.8%), and differences depending on both the population and the clinical status (7 miRNAs, 9.9%). After checking for FDR (*p-adj*), twenty miRNAs resulted to be significantly different between H and PTB (let-7e, miR-10b, miR-127-5p, miR-146a, miR-148a, miR-16, miR-185, miR-192, miR-193a-5p, miR-25, miR-365, miR-451, miR-518d-3p, miR-532-5p, miR-590-5p, miR-660, miR-885-5p, miR-223*, miR-30a, miR-30e). Complete results are available in Table S5.

**Table 1:** Serum miRNAs showing different levels in healthy (H) and pulmonary active tuberculosis (PTB) subjects from the two populations included in the study.

miRNA	Array	TBnew vs TB CHILD (p-val)	H vs PTB (p-val)
has-miR-155-4395459	**A**	0,01375	0,40055
hsa-miR-126-4395339	**A**	0,02580	0,76655
hsa-miR-129-5p-4373171	**A**	0,02600	0,84640
hsa-miR-139-3p-4395424	**A**	0,00975	0,91465
hsa-miR-142-5p-4395359	**A**	0,00025	0,33310
hsa-miR-145-4395389	**A**	<0,00001	0,88630
hsa-miR-146b-5p-4373178	**A**	0,02525	0,10360
hsa-miR-148b-4373129	**A**	0,00170	0,84365
hsa-miR-150-4373127	**A**	0,01065	0,37600
hsa-miR-152-4395170	**A**	<0,00001	0,43940
hsa-miR-17-4395419	**A**	0,00630	0,50405
hsa-miR-184-4373113	**A**	0,00510	0,32595
hsa-miR-195-4373105	**A**	<0,00001	0,16715
hsa-miR-19a-4373099	**A**	0,04110	0,34870
hsa-miR-20b-4373263	**A**	0,00315	0,52460
hsa-miR-220c-4395322	**A**	0,00100	0,29480
hsa-miR-29c-4395171	**A**	0,02855	0,29935
hsa-miR-302c-4378072	**A**	<0,00001	0,55550
hsa-miR-324-3p-4395272	**A**	0,00805	0,75735
hsa-miR-331-3p-4373046	**A**	0,00455	0,43190
hsa-miR-374b-4381045	**A**	0,00745	0,36145
hsa-miR-423-5p-4395451	**A**	0,04475	0,15395
hsa-miR-485-3p-4378095	**A**	<0,00001	0,54070
hsa-miR-574-3p-4395460	**A**	0,04470	0,05040
hsa-miR-597-4380960	**A**	0,00025	0,98915
hsa-miR-628-5p-4395544	**A**	0,01450	0,32615
hsa-miR-744-4395435	**A**	0,00100	0,40245
hsa-miR-872-4395375	**A**	<0,00001	0,06135
hsa-miR-9-4373285	**A**	0,04985	0,21925
MammU6-4395470	**A**	0,00140	0,51640
hsa-miR-135a*-4395343	**B**	0,00020	0,22165
hsa-miR-509-3p-4395347	**B**	0,01160	0,19885
hsa-miR-645-4381000	**B**	0,00005	0,27560
hsa-miR-801-4395183	**B**	<0,00001	0,87435
hsa-miR-923-4395264	**B**	0,00080	0,06465
MammU6-4395470	**B**	0,00045	0,58190
hsa-let-7e-4395517	**A**	0,80890	0,00395*
hsa-miR-10b-4395329	**A**	0,79615	0,0072*
hsa-miR-127-5p-4395340	**A**	0,96665	0,00495*
hsa-miR-130a-4373145	**A**	0,09690	0,02485
hsa-miR-146a-4373132	**A**	0,14045	0,0047*
hsa-miR-148a-4373130	**A**	0,91780	<0,00001*
hsa-miR-16-4373121	**A**	0,17145	0,00045*
hsa-miR-185-4395382	**A**	0,47350	0,00685*
hsa-miR-19b-4373098	**A**	0,42240	0,01720
hsa-miR-24-4373072	**A**	0,29355	0,04625
hsa-miR-25-4373071	**A**	0,18345	0,00065*
hsa-miR-27a-4373287	**A**	0,19845	0,01235
hsa-miR-27b-4373068	**A**	0,12255	0,03670
hsa-miR-342-3p-4395371	**A**	0,77485	0,01620
hsa-miR-365-4373194	**A**	0,48025	0,00345*
hsa-miR-374a-4373028	**A**	0,17845	0,03010
hsa-miR-376c-4395233	**A**	0,32505	0,04740
hsa-miR-451-4373360	**A**	0,50895	0,0082*
hsa-miR-532-5p-4380928	**A**	0,09210	0,00155*
hsa-miR-590-5p-4395176	**A**	0,46820	0,0039*
hsa-miR-660-4380925	**A**	0,48025	0,00095*
hsa-miR-885-5p-4395407	**A**	0,86025	0,003*
RNU48-4373383	**A**	0,90280	0,02350
hsa-miR-144*-4395259	**B**	0,63015	0,02595
hsa-miR-223*-4395209	**B**	0,79420	0,00025*
hsa-miR-30a-4373061	**B**	0,14410	0,00065*
hsa-miR-30a*-4373062	**B**	0,54630	0,03605
hsa-miR-30d-4373059	**B**	0,43060	0,00715
hsa-miR-30e-4395334	**B**	0,50865	0,0012*
hsa-miR-106a-4395280	**A**	0,03535	0,01145
hsa-miR-125a-5p-4395309	**A**	0,00380	0,03295
hsa-miR-192-4373108	**A**	0,04850	0,001*
hsa-miR-193a-5p-4395392	**A**	0,01675	0,00485*
hsa-miR-212-4373087	**A**	<0,00001	0,02215
hsa-miR-483-5p-4395449	**A**	0,00120	0,03800
hsa-miR-518d-3p-4373248	**A**	0,03350	0,00155*

* miRNAs showing a p-adj <0.05.

### Pools *vs* Individuals

**Figure 5:**
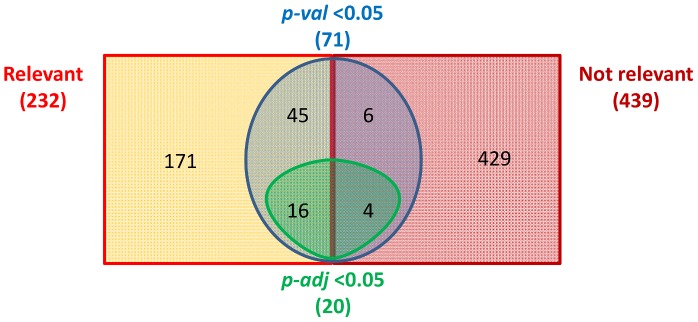
Analysis of the results of serum miRNAs in healthy controls (H) and pulmonary active tuberculosis (PTB) using pooled and individual specimens. Squares summarize results obtained by comparison of miRNAs in pooled specimens, whereas circles define results from individual sera.

Out of the 20 miRNAs showing a significant *p-adj* in the study on individuals (Table 1), 16 (80%) had already been identified as “relevant miRNA” in the analysis of pooled specimens (Figure 5). Among those miRNAs, nine showed differences between H and PTB pools in each group; four showed differences only in *TB CHILD* pooled specimens, whereas three only in the pools from *TBnew* group. Four miRNAs with a significant *p-adj* in the study on individuals had not been detected as relevant by the first screening on pooled specimens. Figure 5 shows that the analysis on pools correctly excluded 429 out of 439 “not relevant” miRNAs from further investigations.

Then, for the 16 miRNAs found to be relevant in the pooled specimens analysis and significant (*p-adj* <0.05) in individual sera (let-7e, miR-146a, miR-148a, miR-16, miR-192, miR-193a-5p, miR-25, miR-365, miR-451, miR-532-5p, miR-590-5p, miR-660, miR-885-5p, miR-223*, miR-30a), we compared the direction of the variation (increase or decrease) in pooled and single PTB serum analyses. From this comparison we found that some miRNAs showed inconsistent results between individual and pooled specimens (Table 2): eleven miRNAs showed the same type of variation in individual and pooled samples across both study groups considered, three miRNAs showed discordant variation only in *TBnew* or *TB CHILD* population, and one showed discordant variation in both *TBnew* and *TB CHILD* groups. Five and three targets were found to be associated only to the *TB CHILD* and to the *TBnew* group, respectively. In conclusion, from the combined analysis on pools and individuals, a total of 15 miRNAs were identified as a signature for discriminating between H and PTB (let-7e, miR-146a, miR-148a, miR-16, miR-192, miR-193a-5p, miR-25, miR-365, miR-451, miR-532-5p, miR-590-5p, miR-660, miR-885-5p, miR-223*, miR-30e).

**Table 2:** Serum miRNA levels in pulmonary active tuberculosis (PTB) subjects as compared with healthy controls (H).

	*TBnew*			*TB CHILD*			Combined				
miRNAs p-adj <0.05	Individuals	Pools		Individuals	Pools		Individuals	Pools		Relevance in pooled specimens	To be considered for
hsa-let-7e-4395517	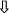	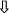		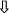	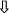		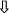	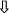		*TBnew, TB CHILD*	*TBnew, TB CHILD*
hsa-miR-146a-4373132	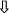	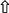	×	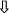	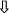		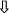	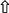		*TB CHILD* only	*TB CHILD only*
hsa-miR-148a-4373130	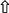	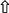		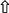	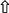		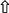	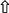		*TBnew, TB CHILD*	*TBnew, TB CHILD*
hsa-miR-16-4373121	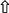	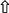		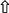	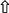		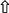	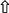		*TBnew only*	*TBnew only*
hsa-miR-192-4373108	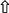	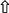		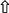	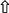		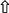	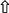		*TBnew, TB CHILD*	*TBnew, TB CHILD*
hsa-miR-193a-5p-4395392	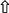	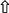		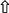	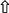		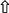	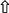		*TBnew, TB CHILD*	*TBnew, TB CHILD*
hsa-miR-25-4373071	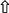	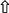		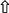	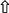		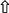	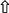		*TBnew only*	*TBnew only*
hsa-miR-365-4373194	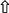	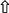		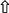	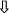	×	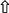	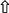		*TBnew* only	*TBnew only*
hsa-miR-451-4373360	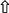	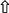		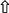	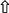		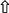	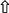		*TBnew, TB CHILD*	*TBnew, TB CHILD*
hsa-miR-532-5p-4380928	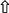	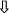	×	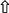	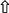		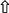	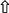		*TB CHILD only*	*TB CHILD only*
hsa-miR-590-5p-4395176	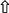	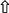		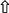	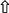		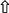	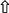		*TBnew, TB CHILD*	*TBnew, TB CHILD*
hsa-miR-660-4380925	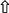	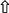		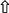	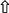		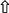	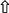		*TB CHILD only*	*TB CHILD only*
hsa-miR-885-5p-4395407	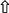	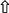		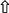	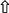		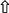	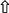		*TBnew, TB CHILD*	*TBnew, TB CHILD*
hsa-miR-223*-4395209	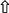	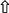		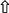	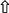		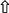	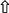		*TB CHILD* only	*TB CHILD only*
hsa-miR-30a-4373061	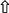	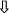	×	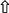	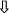	×	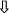	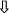	×	*TBnew, TB CHILD*	*–*
hsa-miR-30e-4395334	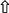	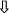	×	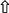	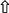		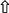	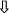	×	*TBnew, TB CHILD*	*TB CHILD only*

The table reports data from individual and pooled specimens. Discrepancies are marked by *x*.

### Diagnostic Performances of the Identified Serum miRNA Signature

**Figure 6:**
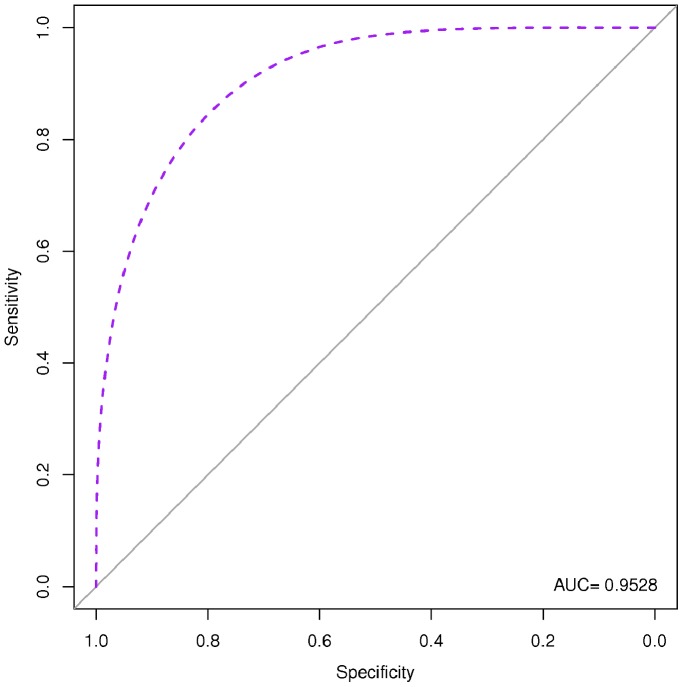
Receiver Operating Characteristic (ROC) based on Akaike information criterion (AIC) logistic regression for the 15-miRNA signature. AUC: area under the curve. The AIC model identified as the best performing miRNA signature the following: miR-let-7e, miR-146a, miR-16, miR-25, miR-365, miR-451, miR-885-5p, miR-223*.

**Figure 7:**
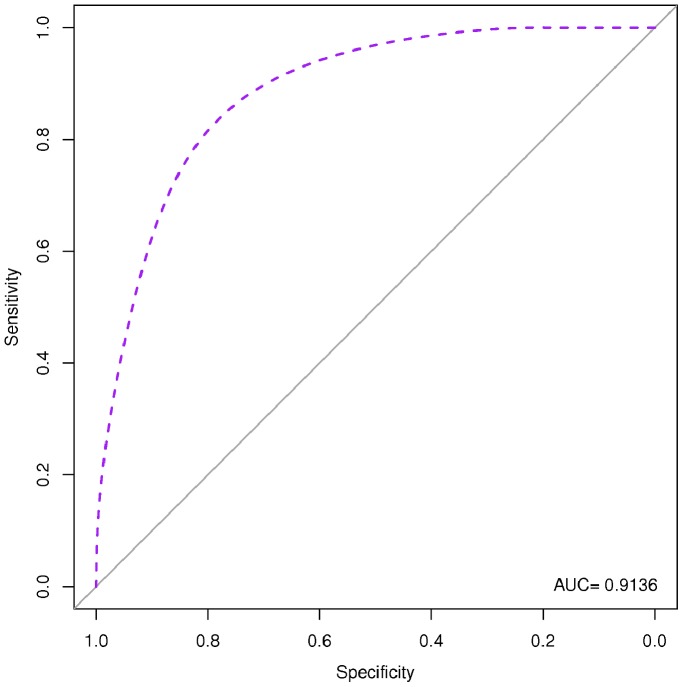
Receiver Operating Characteristic (ROC) based on Akaike information criterion (AIC) logistic regression for the 10-miRNA signature specific for *TBnew* population. AUC: area under the curve. The AIC model identified as the best performing miRNA signature the following: miR-let-7e, miR-192, miR-25, miR-451.

**Figure 8:**
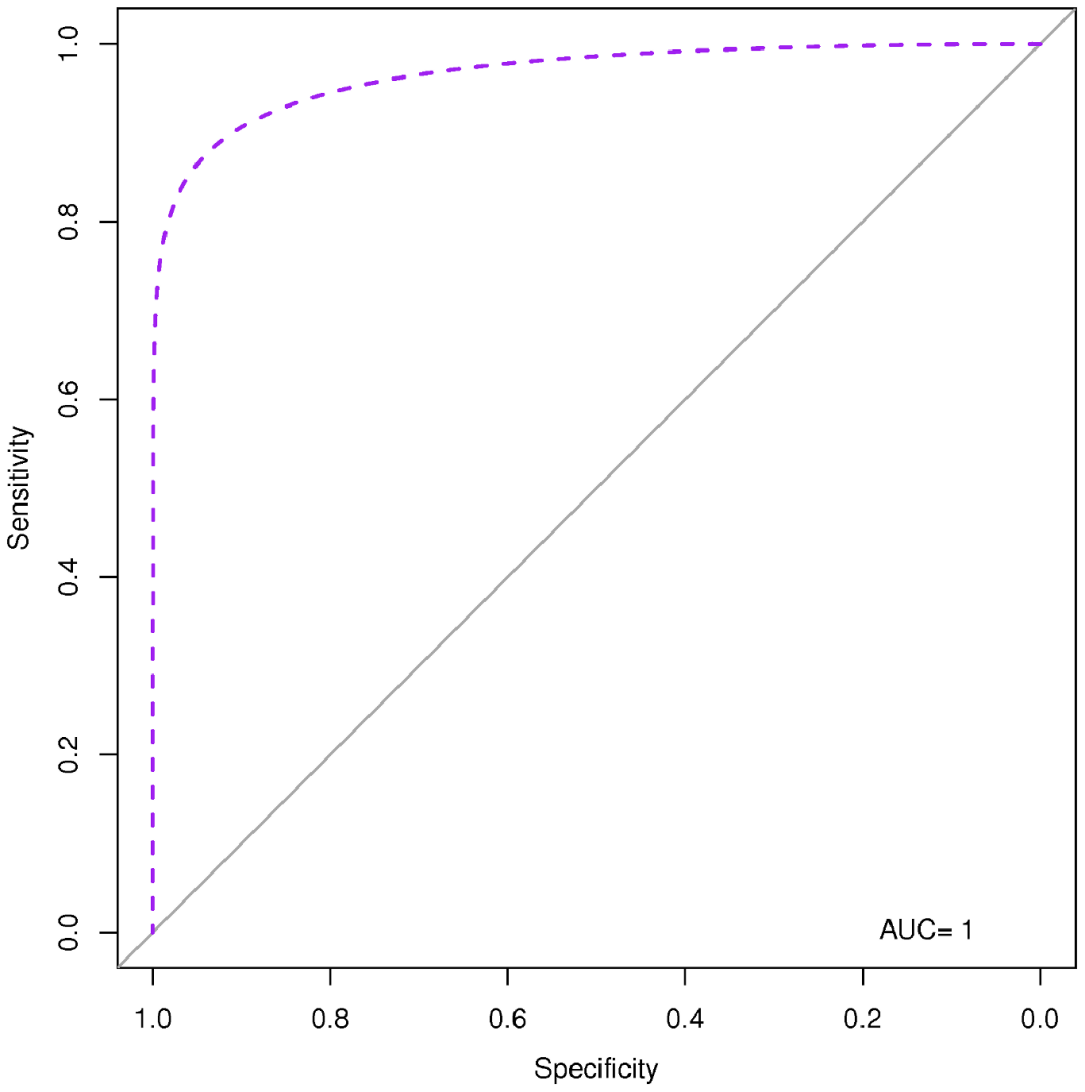
Receiver Operating Characteristic (ROC) based on Akaike information criterion (AIC) logistic regression for the 12-miRNA signature specific for *TB CHILD* population. AUC: area under the curve.

We used two approaches to evaluate the diagnostic performances of the identified serum miRNA signature: RVM model, and AIC logistic regression analysis. Both were cross-validated by the use of a LOOCV approach. All fifteen miRNAs (let-7e, miR-146a, miR-148a, miR-16, miR-192, miR-193a-5p, miR-25, miR-365, miR-451, miR-532-5p, miR-590-5p, miR-660, miR-885-5p, miR-223*, miR-30e) were used to re-classify the 56 subjects analyzed as single sera obtaining a diagnostic accuracy of 82% by RVM, and of 77% by logistic regression. The diagnostic accuracy increased up to 83% by RVM and 81% by logistic regression when miRNAs identified as “*TBnew*-specific” (let-7e, miR-148a, miR-16, miR-192, miR-193a-5p, miR-25, miR-365, miR-451, miR-590-5p, miR-885-5p) re-classify *TBnew* single serum samples. A similar increase was observed when the “*TB CHILD*-specific” signature (let-7e, miR-146a, miR-148a, miR-192, miR-193a-5p, miR-451, miR-532-5p, miR-590-5p, miR-660, miR-885-5p, miR-223*, miR-30e) was used on the 10 individual samples from *TB CHILD* group (95% by RVM, and 100% by logistic regression, respectively). Table 3 and Table 4 summarize the diagnostic performances of the miRNA signatures achieved by the different approaches used for the classification of subjects. AUCs for the regression logistic analyses are reported in Figure 6, Figure 7, and Figure 8.

**Table 3:** Diagnostic performances of the serum miRNA signatures in the Relevance Vector Machine (RVM).

Serum miRNA signature (n)	All (15)	TBnew (10)	TB CHILD (12)
**n of individuals (H/PTB)**	56 (28/28)	36 (18/18)	20 (10/10)
**Sensitivity % (95% CI)**	85.71 (68.51–94.30)	77.78 (54.78–91.00)	100.00 (72.25–100.00)
**Specificity % (95% CI)**	78.57 (60.46–89.79)	88.89 (67.20–96.90)	90.00 (59.58–98.21)
**PPV % (95% CI)**	80.00 (62.69–90.50)	87.50 (63.98–96.50)	90.91 (62.26–98.38)
**NPV % (95% CI)**	84.62 (66.47–93.85)	80.00 (58.40–91.93)	100.00 (70.08–100.00)
**Diagnostic accuracy % (95% CI)**	82.14 (70.16–90.00)	83.33 (68.11–92.13)	95.00 (76.39–99.11)
**Likelihood ratio of a positive test % (95% CI)**	4 (2.846–5.621)	7 (2.524–19.41)	10 (1.409–70.99)
**Likelihood ratio of a negative test % (95% CI)**	0.1818 (0.1087–0.3041)	0.25 (0.1508–0.4144)	0

The Table reports diagnostic performances already corrected for the leave-one-out cross validation (LOOCV).

**Table 4:** Diagnostic performances of the serum miRNA signatures in the AIC logistic regression model.

Serum miRNA signature (n)	All (15)	TBnew (10)	TB CHILD (12)
**n of individuals (H/PTB)**	56 (28/28)	36 (18/18)	20 (10/10)
**Sensitivity % (95% CI)**	71.43 (52.94–84.75)	72.22 (49.13–87.50)	100.00 (72.25–100.00)
**Specificity % (95% CI)**	82.14 (64.41–92.12)	88.89 (67.20–96.90)	100.00 (72.25–100.00)
**PPV % (95% CI)**	80.00 (60.87–91.14)	86.67 (62.12–96.26)	100.00 (72.25–100.00)
**NPV % (95% CI)**	74.19 (56.75–86.30)	76.19 (54.91–89.37)	100.00 (72.25–100.00)
**Diagnostic accuracy % (95% CI)**	76.79 (64.23–85.90)	80.56 (64.97–90.25)	100.00 (83.89–100.00)
**Likelihood ratio of a positive test % (95% CI)**	4 (2.599–6.156)	6.5 (2.302–18.35)	undefined
**Likelihood ratio of a negative test % (95% CI)**	0.3478 (0.2672–0.4527)	0.3125 (0.2079–0.4696)	0

The Table reports diagnostic performances already corrected for the leave-one-out cross validation (LOOCV).

## Discussion

In TB biomarker research, published studies on highly multiplexed assays focus on proteomics, gene expression, transcriptomics, and miRNAs, as predictors of disease, disease recurrence or drug resistant infection [38]–[47]. Our study on serum miRNA signatures ascertains the value of these biomarkers for TB disease classification, as previously reported by others [21]–[23]. However, previous studies did not consider the impact of the genetic makeup on the miRNA signatures: ethnicity, together with age and gender, could influence the levels of circulating miRNAs [48]. By the comparison of two populations with different genetic makeup we showed that some population-specific miRNAs can increase the diagnostic accuracy for active TB.

While much research is still focused on assessing the quality of single biomarkers, there is an emerging interest in panels of biomarkers composed of multiple candidate targets which are neither specific nor sensitive when used as single tests, but which show a good performance when used in combination. In this study we used both RVM and logistic regression methods supported by a LOOCV approach to evaluate the diagnostic performances of serum miRNAs identified as a signature rather than as single biomarkers. The presence of miRNAs detectable in at least the 80% of one category indicates a higher diagnostic index; moreover, our filtering approach did not introduce biases in detecting potential on/off miRNAs between categories. Indeed, whenever a serum sample is tested for miRNA, its diagnostic relevance will be attested by its level rather than by their presence/absence in H and PTB categories.

To ascertain if a serum miRNA signature could discriminate between different categories of patients, we used a restrictive stratification screening approach to minimize the number of possible biases. Despite subjects within the H category showed a mean age lower than the other categories, we consider this to be only a minor drawback of the study. Particular attention should be reserved to specific subgroups (*e.g.* childhood and elderly) where age would expect to have a bigger impact. Another possible drawback could be the heterogeneity in terms of genetic background (estimated on the basis of the country of birth) in the *TBnew* group. This could partially explain lower performances of the miRNA signature on this population. The results on pooled specimens identified differences in serum miRNA profiles in the categories analyzed. Interestingly, according to our qualitative analysis based on the Empirical Distribution Function of residuals, serum miRNAs would allow not only to discriminate LTBI and PTB, but also PTB from OPI and EPTB. Indeed, the 134 miRNAs showing relevant differences in serum level of LTBI and PTB subjects include a smaller subset of miRNAs that could be used as specific signature to discriminate between these two categories. A similar approach could be applied for the 132 miRNAs showing relevant differences in serum levels of PTB and OPI subjects and for the 124 miRNAs differentiating PTB and EPTB subjects. Further studies on cohorts of LTBI, OPI and EPTB individuals will allow to identify specific miRNA patterns and to evaluate their diagnostic accuracy. The discriminatory power of serum miRNAs observed is further supported by the fact that the same findings have been confirmed in the two different groups (namely *TBnew* and *TB CHILD*) for the signature identified for the comparison H-PTB. Additionally, the use of pooled specimens allowed halving the number of targets to be analyzed by excluding miRNAs under the detection threshold or showing very little changes across categories.

To refine our findings, we performed serum miRNA analysis on individual sera from H and PTB subjects. Table 5 summarizes the comparison between our results and previously published studies. In the first study on circulating miRNAs as biomarkers for TB reported by Fu and colleagues [21], it was demonstrated that 92 miRNAs had significantly different levels in the sera of healthy controls *vs* PTB subjects: 59 miRNAs were down-regulated and 33 miRNAs were up-regulated in the serum of TB patients. One-by-one comparison is not possible due to different analytical platforms and normalization strategies, but some homologies between the study by Fu and our results can be observed. For example, miRNAs belonging the families let-7, miR-30, and miR-146 were found to be significantly different between H and PTB in both studies. miRNAs miR-590-5p, miR-185, miR-660, let-7e, miR-25, miR-146a, and miR-885-5p showed to be differentially expressed between healthy controls and PTB subject also in the study reported by Qi and colleagues [22]. miR-197 which was observed to be slightly increased in our study was also reported to be increased in sera from pulmonary TB patients by Abd-El-Fattah and colleagues [23]. However, the previous studies did not consider the genetic background of the subjects enrolled. Differently, in the present study the inclusion of groups with different genetic background allowed us to better define serum miRNA signatures associated to a different (health) status. Indeed, subjects belonging to the same status (i.e. H or PTB) showed different serum miRNA levels between the *TBnew* and the *TB CHILD* groups in pooled specimens (Table S4). Comparing individual sera from subjects belonging to the two populations we found significant differences in the level of several miRNAs (Table S5). Despite larger population-based studies are still needed, our data support the hypothesis that the genetic background could influence the specific serum miRNA profiles. Interestingly, by matching miRNA signatures from pooled specimens, individual specimens, and direction of variation (increase/decrease) we identified 7 common discriminatory miRNAs (let-7e, miR-148a, miR-192, miR-193a-5p, miR-451, miR-590-5p, miR-885-5p) plus three miRNAs specific for the *TBnew* group (miR-16, miR-25, miR-365), and five miRNAs specific for the *TB CHILD* group (miR-146a, miR-532-5p, miR-660, miR-223*, miR-30e). The diagnostic accuracy for each single miRNA was found to be <75% (data not shown), while better results were achieved by using the approach of “signatures”: AUC values were above 0.90 and the use of the entire fifteen-miRNAs signature provided a diagnostic accuracy between 77% and 82% in a LOOCV approach (logistic regression and RVM, respectively). Population-specific signatures allowed to further improve classification accuracy (81–83% for *TBnew*, and 95–100% for *TB CHILD*, respectively). As mentioned before, serum miRNA signatures showed less efficiency in classifying subjects belonging to the *TBnew* group. Our hypothesis is that, despite the mild to moderate differences, the genetic background heterogeneity of this group is likely affecting the classification performances of the miRNA signature. The higher number of miRNAs with discrepancy variations (in terms of increase/decrease) between individual and pooled specimens provides some evidence on the heterogeneity of the *TBnew* population.

**Table 5:** Comparison between the serum miRNA signature found in the present and the miRNAs reported in the previous studies.

miRNA	Fu et al, 2011	Qi et al, 2012
hsa-let-7e-4395517	let-7 family	let-7e, let-7 family
hsa-miR-146a-4373132	miR-146a	miR-146a
hsa-miR-148a-4373130	–	–
hsa-miR-16-4373121	–	–
hsa-miR-192-4373108	–	–
hsa-miR-193a-5p-4395392	–	miR-193 family
hsa-miR-25-4373071	–	miR-25
hsa-miR-365-4373194	miR-365 family	–
hsa-miR-451-4373360	–	–
hsa-miR-532-5p-4380928	–	miR-532 family
hsa-miR-590-5p-4395176	–	miR-590-5p
hsa-miR-660-4380925	–	miR-660
hsa-miR-885-5p-4395407	–	miR-885-5p
hsa-miR-223*-4395209	–	miR-223 family
hsa-miR-30e-4395334	miR-30 family	miR-30 family

As for the function of extracellular miRNAs, current evidences suggest a regulatory role on the expression of target genes when taken up by recipient cells, with the peculiar capability to act on several targets at a time and to operate in a network with other extracellular/intracellular miRNAs [17], [19]. A “hormonal” role to extracellular miRNAs was attributed [17], [19]. If this interpretation proves to be true, we could then be facing a major advancement in modern biology not only for better understanding of biological complexity, but also in terms of diagnostic and even therapeutic possibilities [49]. Target cells for extracellular miRNAs and related targeting mechanism are poorly understood, thus careful interpretation of circulating miRNA origin and function should be considered.

Here we described a serum miRNA signature discriminating H and PTB subjects. Despite promising results, several challenges in pre-analytical and analytical phases remain in the analysis of circulating miRNAs. Accurate large-cohort studies are therefore required to validate PTB-specific miRNA signatures, and to identify miRNA signatures also for LTBI, EPTB and pathologies (like pneumonia, cancer, HIV infection and sarcoidosis) often in differential diagnosis with TB. The inclusion of different sets of biomarkers (*e.g.* cytokines, antibodies) could also help in achieving higher discrimination power amongst the closest categories.

## Supporting Information

Table S1 **Details on subjects included in pooled and individual specimens analyzed in the study.** H: healthy controls; LTBI: latent tuberculosis infection; PTB: pulmonary active tuberculosis; EPTB: active extra-pulmonary tuberculosis; OPI: other pulmonary infections; PTB/HIV: PTB in HIV-positive. SD: standard deviation.
(XLSX)

Table S2 **Comparison of the C_t_ values among the four miRNAs detected in both array A and array B types between different populations (** ***TBnew*** **,** ***TB CHILD*** **) and different specimens (** ***individual sera*** **,** ***pooled sera*** **).** Mean C_t_ values are reported together with the standard deviation after quantile normalization.
(XLSX)

Table S3 **Normalized C_t_ values obtained from individual and pooled specimens after quantile normalization.**
(XLSX)

Table S4 **Complete data analysis on pooled specimens.** The Table reports residual values obtained by each comparison performed between the categories. Relevant differences are highlighted.
(XLSX)

Table S5 **Complete data analysis on individual specimens.** The Table reports the p-value for the following comparison on individual specimens: *TBnew vs TB CHILD*, and healthy control (H) *vs* pulmonary active tuberculosis (PTB). P-adjusted (*p-adj*) is reported for the comparison H *vs* PTB. Values <0.05 are highlighted. na: not applicable (miRNAs not detected in at least 80% of subjects in at least one of the category considered where not included in the analysis).
(XLSX)
